# Supplementary material for: Feeding-dependent tentacle development in the sea anemone Nematostella vectensis
Source: Nat Commun. 2020 Sep 2;11:4399. doi: 10.1038/s41467-020-18133-0 (PMC7467937; doi:10.1038/s41467-020-18133-0)
Supplement: Supplementary file 1 — Supplementary Information [file 41467_2020_18133_MOESM1_ESM.pdf]

Feeding-dependent tentacle development in the sea anemone *Nematostella vectensis*

Aissam Ikmi, Petrus J. Steenbergen, Marie Anzo, Mason R. McMullen, Anniek Stokkermans, Lacey R. Ellington, and Matthew C. Gibson

Supplementary Figures 1-12

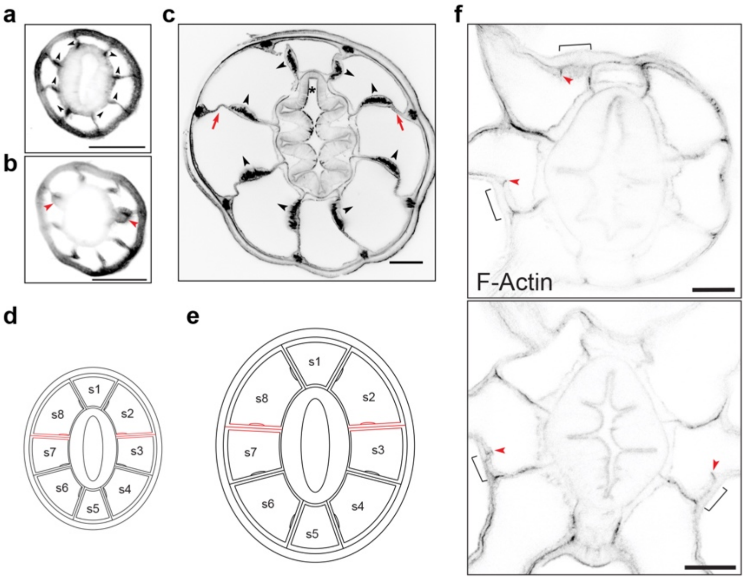

Supplementary Figure 1: Body segments in primary and adult polyps. **a** Cross-section through the oral pole at the base of the tentacles in a primary polyp (n=10 polyps, two independent experiments). **b** Cross-section through the oral pole of the same animal at the base of the pharynx and showing the most apical domain of primary mesenteries (red arrowheads). **c** Cross-section through the oral pole of an adult polyp (n=6 polyps, two independent experiments). Red arrows indicate the two primary mesenteries. Black asterisk (*) indicates the position of the siphonoglyph. All animals are labelled with Phalloidin (F-actin, in grey). Each mesentery bears a retractor muscle. The orientation of these muscles (black arrowheads) can serve as a readout for the polarity of the directive axis. **d**, **e** Diagrammatic cross-section through the oral pole showing the eight body segments in primary and adult polyps, respectively. Primary mesenteries are colored in red. **f** Confocal cross-sections of representative oral poles bearing eight tentacles and stained to label F-actin (n=6 polyps, two independent experiments). The formation of short gastrodermis folds within segments precedes the third and fourth trans-budding events. Red arrowheads indicate new endodermal folds enriched in F-actin. Brackets show new tentacle territories. Scale bars are 100μm in **a-c** and 50μm in **f**.

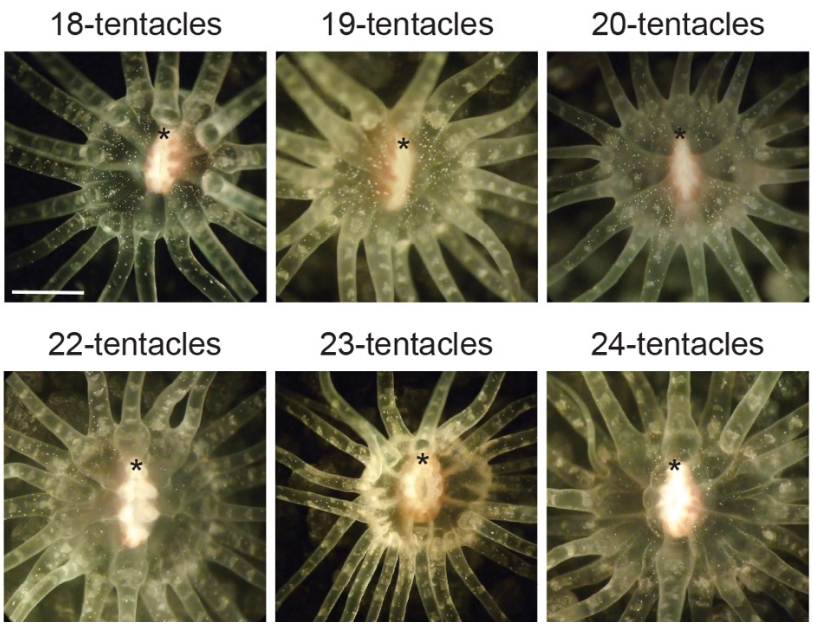

Supplementary Figure 2: **Adult polyps bearing more than 16-tentacles**. Oral views of adult polyps (polyps with 18-tentacles n=22, 19-tentacles n=3, 20-tentacles n=16, 22-tentacles n=8, 23-tentacles n=3, 24-tentacles n=1, two independent experiments). Black asterisk (*) indicates the position of the siphonoglyph. Scale bar is 1mm.

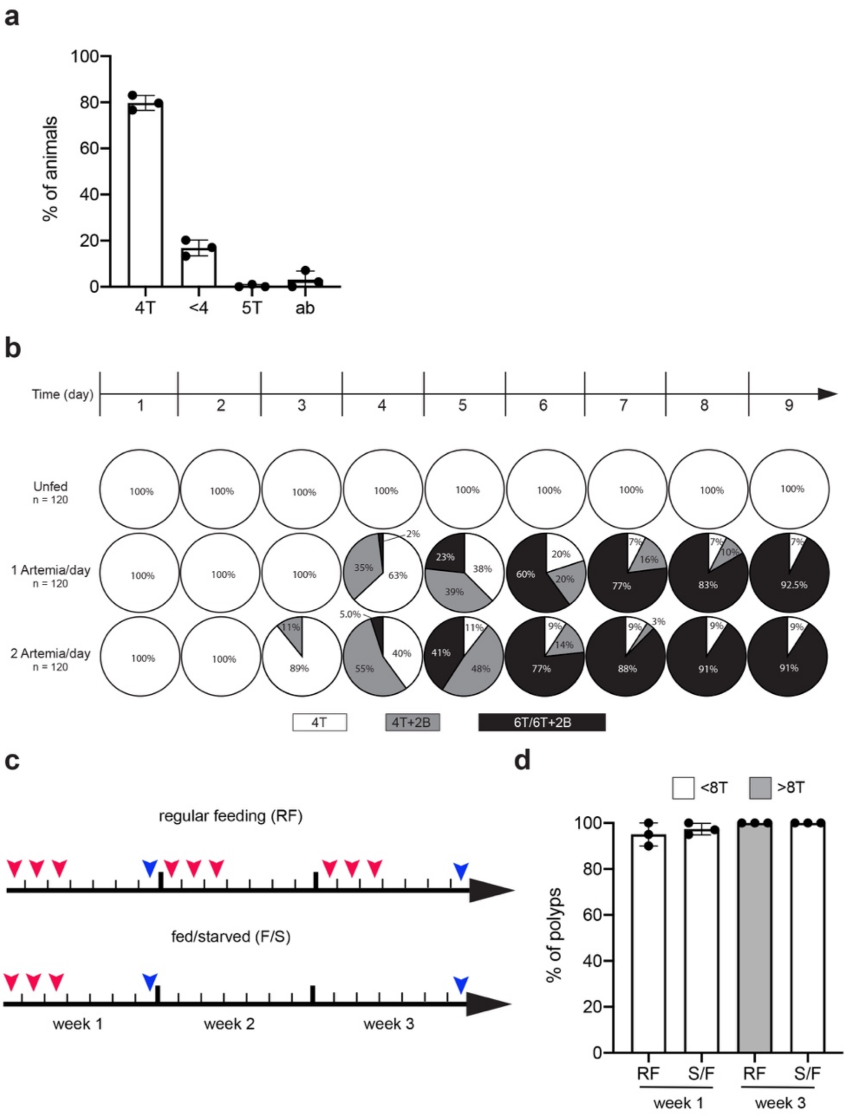

Supplementary Figure 3: Feeding-dependent tentacle addition. **a** Tentacle number in unfed primary polyps in three independent experiments (n=101, n=105 and n=100 polyps). Note that the frequent number of tentacles in unfed primary polyps is four. Polyps with 4-tentacles (4T), polyps with more than 4 tentacles (>4T), polyps with less then 4 tentacles (<4T), abnormal animals (ab). **b** Feeding is required for subsequent tentacle addition in the polyp stage (n=120 polyps for each condition). Unfed primary polyps did not add tentacles. Polyps were hand-fed with 1 or 2 *Artemia* per day. Polyps fed twice developed buds earlier than those fed once per day. **c** Tentacle addition in polyps that were fed for three weeks (regular feeding, RF) versus those that were fed for one week and then starved for two weeks (fed/starved, R/S). Red arrowheads indicate the feeding days. Blue arrowheads show the days where the tentacles were counted. **d** Quantification of tentacle number in polyps exposed to these feeding treatments. Each dot represents an independent experiment. Data are mean ± SD for error bars. Source data are provided as a Source Data file.

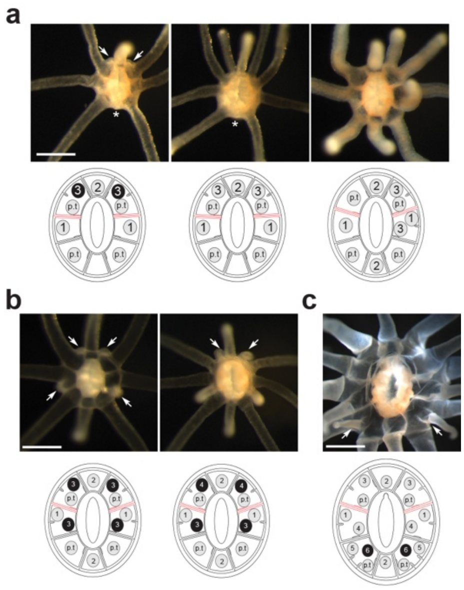

Supplementary Figure 4: Variability in tentacle addition. a Asynchrony in the development of tentacle pair (n=155 polyps, three independent experiments). **b** Two pairs of buds formed simultaneously instead of sequentially (n=66 polyps, three independent experiments). **c** A polyp showing a trans-budding event during phase III of tentacle addition (n=6 polyps, three independent experiments). White asterisk indicates a missing tentacle and white arrows show tentacle buds. Scale bar are 250μm in **a**-**b** and 500μm in **c**.

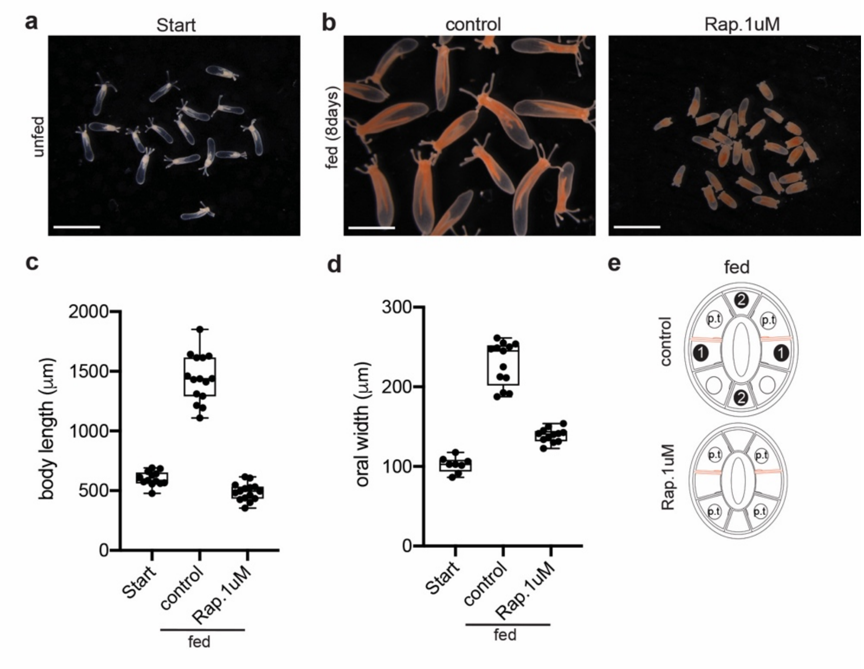

Supplementary Figure 5: TOR-dependent growth and budding in fed polyps. a Unfed primary polyps (start). **b** Control and Rapamycin (Rap.) treated polyps that were fed for 8 days in two independent experiments. Note the orange color of fed polyps compared to unfed. Scale bars are 1mm. **c**, **d** Quantification of body length and oral width of indicated conditions (start, n=13 polyps; control n=15 polyps; Rap. 1µM n=15 polyps). Data are shown as individual data points for each polyp in a Box and Whiskers graph (bottom: 25%; top: 75%; line: median; whiskers: min to max). **e** Diagrams showing tentacle arrangement in fed controls and Rapamycin treated animals. Source data are provided as a Source Data file.

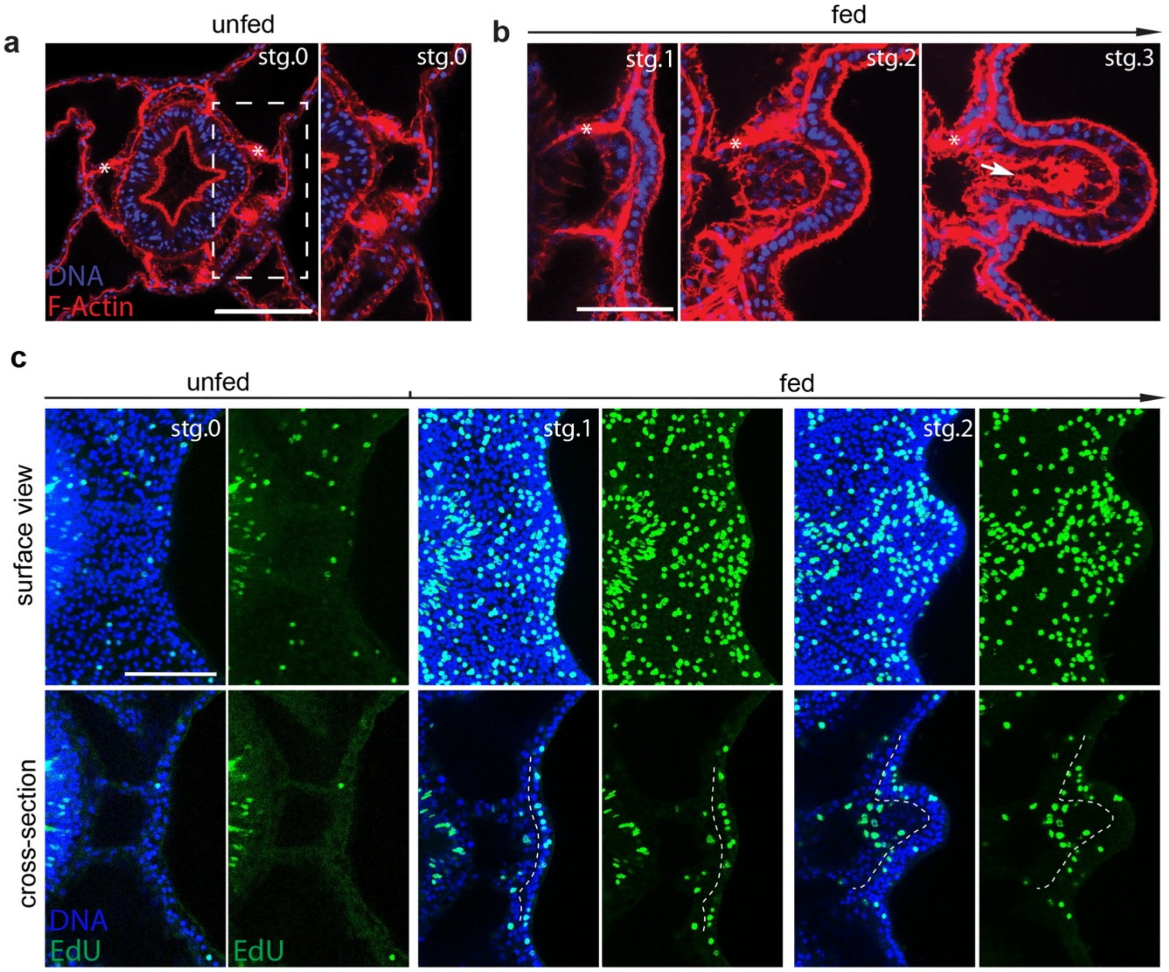

Supplementary Figure 6: Staging of tentacle buds in fed polyps. **a**, **b** Confocal z-section of the oral pole of indicated polyps stained with phalloidin to visualize F-Actin (red) and Hoechst to label nuclei (blue) in three independent experiments. Inset box in (**a**) is a zoom-in of the segment 3. **b** Confocal sections of developing buds at sequential stages. Budding initiates from a flat epithelial architecture (stage 0, stg.0 n=12 polyps) that undergoes a slight asymmetric thickening towards the primary mesentery (stage 1, stg.1 n=10 polyps) followed by the formation of an outgrowth (stage 2, stg.2 n= 14 polyps). A mature bud (stage 3, stg.3 n=16 polyps) is formed when a lumen appears (white arrow). **c** Surface views and cross-sections of s3 segments in animals stained for EdU incorporation (green) and with Hoechst (blue) to visualize S-phase cells and nuclei at the indicated feeding conditions (two independent experiments). Scale bars are 50μm in **a-b** and 25μm in **c**.

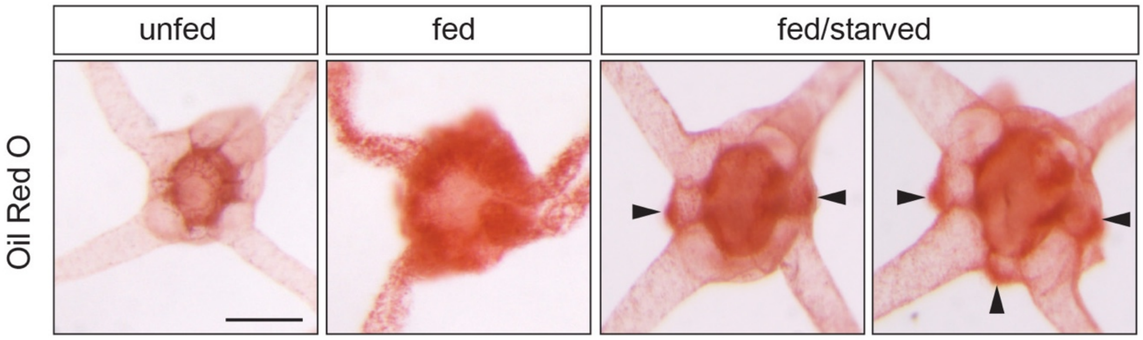

Supplementary Figure 7: Enrichment of lipid droplets in tentacle primordia. Oral views of animals stained with Oil Red O at the indicated feeding conditions (unfed n=8 polyps, fed n=4 polyps, fed/starved n=20 polyps, two independent experiments). Arrowheads indicate the enrichment of Oil Red O staining in tentacle primordia. Scale bar is 50µm.

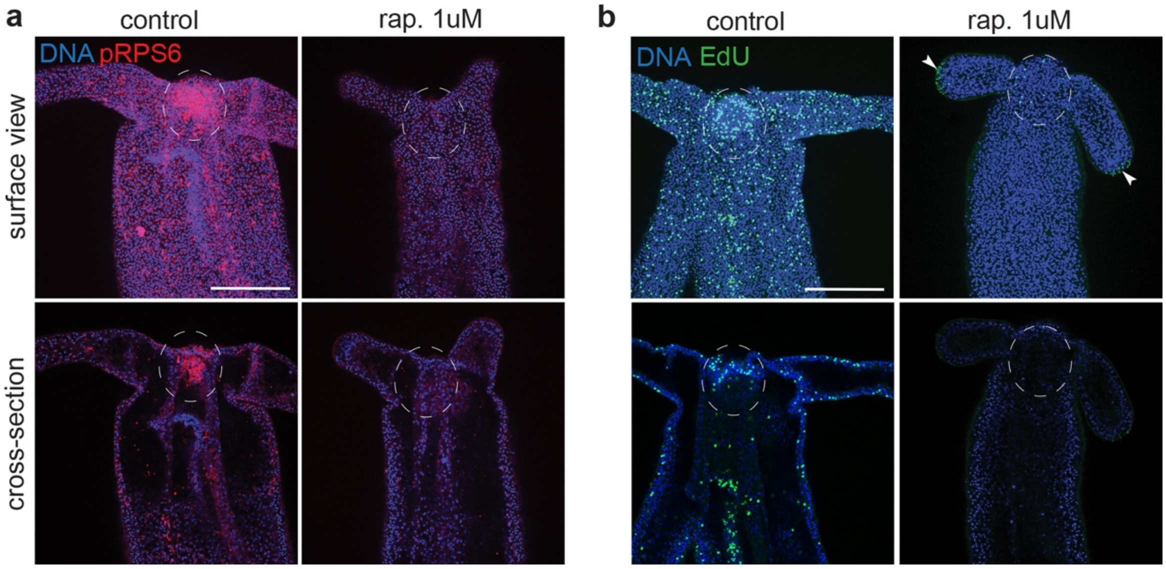

Supplementary Figure 8: **TOR controls global and bud-localized cell proliferation. a** Confocal projection of lateral views. Control (n=12 polyps) and Rapamycin-treated polyps (n=10 polyps) were fed for 4 days and then stained with an antibody against pRPS6 (red) and Hoechst to label nuclei (blue) (two independent experiments). Rapamycin was added at day 3 and incubated for 24 hours. **b** Confocal projections of lateral views of 4 days fed controls (n=6 polyps) and Rapamycin-treated polyps (n=8 polyps) stained for EdU incorporation (green) and Hoechst to label nuclei (blue) (two independent experiments). Dashed circles indicate the location of tentacle primordia in the outer and inner layers. Scale bars are 50μm.

```
Human           MKLNISFPATGCQKLIEVDDERKLRTFYEKRMATEVAADALGEEWKGYVVRISGGNDKQG 60
Nematostella    MKLNISYPVTGCQKLIEIEDERKVRSFYDKRMAMEVSGECLGDEWKGYVFRITGGNDKQG 60
Yeast           MKLNISYPVNGTQKTIEVDDEHRVRVFYDKRIGQEVNGEAVGDEFKGYVFKIAGGNDKQG 60
                ******:*..* ** **::**:::* **:**:. ** .:.:*:*:****.:*:*******

Human           FPMKQGVLTHGRVRLLLSKGHSCYRPRRTGERKRKSVRGCIVDANLSVLNLVIVKKGEKD 120
Nematostella    FPMKQGIMTNGRVRLLLSKGHSCYRPRRTGERKRKSVRGCIVDSQLSVLSLVIVKKGEQD 120
Yeast           FPMKQGVLLPTRVKLLMAKGTSCYRPRRNGERKRKSVRGAIVGPDLAVLALIITKKGDQE 120
                ******::   **:**::** *******.**********.**..:*:** *:*.***:::

Human           IPGLTDTTVPRRLGPKRASRIRKLFNLSKEDDVRQYVVRKPLN-KEGKKPRTKAPKIQRL 179
Nematostella    IPGLTDNTIPRRLGPKRVGKIRKMFNLSKEDDVRQYVIRRPLPEKEGKKAKSKAPKIQRL 180
Yeast           IEGITNESVPKRLGPKRANNIRKFFGLTKDDDVRDFVIRREVV--KGDKTYTKAPKIQRL 178
                * *:*: ::*:******...***:*.*:*:****::*:*: :   :*.*. :********

Human           VTPRVLQHKRRRIALKKQRTKKNKEEAAEYAKLLAKRMKEAKEKRQEQIAKRRRLSSLRA 239
Nematostella    VTPVVLQRKRKRLALKRQRAQKCKQEAADYAKLLAKRAKEAKEKRHEQLMKKRRASSLRD 240
Yeast           VTPQRLQRKRHQRALKVRNAQAQREAAAEYAQLLAKRLTEKKAEKAE--ERKRRASSLKA 236
                ***  **:**:: *** :.::  :: **:**:***** .* * :: *   ::** ***:

Human           STSKSESSQK 249
Nematostella    SVSAK----- 245
Yeast           ----------
```

Supplementary Figure 9: Protein sequence alignment of ribosomal protein S6. Protein sequences are from human, *Nematostella* and yeast. Asterisks (*) show conserved residues. Colons and periods indicate conservation of groups of strongly and weakly similar properties, respectively. The phosphorylated serines in human are conserved and highlighted in green.

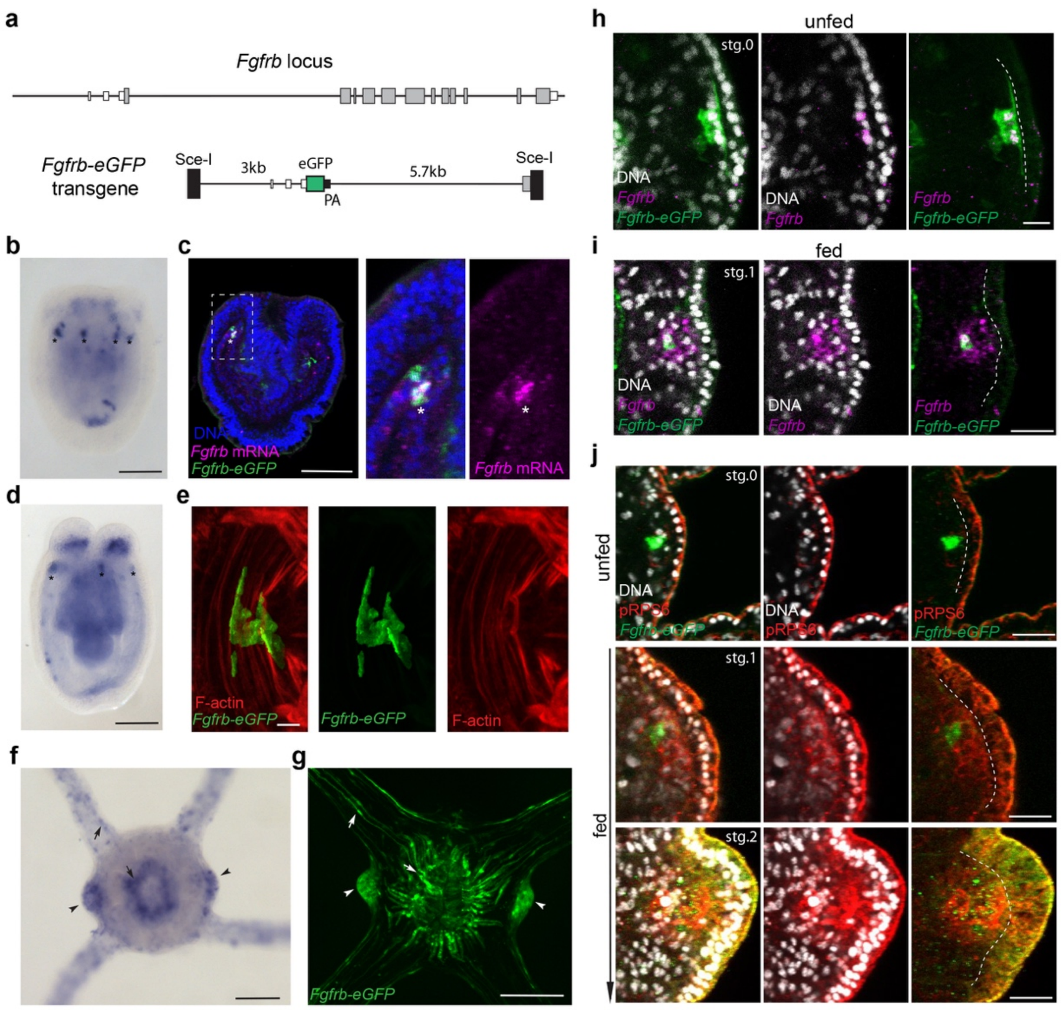

Supplementary Figure 10: ***Fgfrb* expression marks tentacle primordia in fed polyps. a** Gene model of the *Fgfrb* locus and map of the *Fgfrb-eGFP* transgene. **b**, **c, f** *In situ* hybridization of *Fgfrb* in developing *Nematostella* (two independent experiments). **b** Planula (n=8 animals). **d** Unfed polyp (n=6 animals). **f** Oral views of a fed polyp (n=4 animals). Asterisks indicate clusters of *Fgfrb*-expressing cells in the gatrodermis. **c** Confocal z-projections of the *Fgfrb-eGFP* transgenic planula (n=4 animals) stained with α-eGFP (green) and Hoechst (blue) as well as labeled for *Fgfrb* mRNA (purple) (two independent experiments). Inset box is a zoom view of panel C. **e** Confocal z-projections of the *Fgfrb-eGFP* transgenic polyp stained with α-eGFP (green) and Phalloidin (red) showing a high magnification of an *Fgfrb*-positive cell (n= 20 cells, two independent experiments). Scale bar is 3μm. **g** Confocal z-projections of a budded *Fgfrb-eGFP* polyp (n=20 polyps, two independent experiments). Arrowheads indicate tentacle buds. Arrows shows longitudinal muscle cells. **h, i, j** Panels from Figure 6c-e showing nuclear staining. White dashed lines separate the two body layers. Scale bars are 50μm in **d**, **c, d**, **f** and **g**.

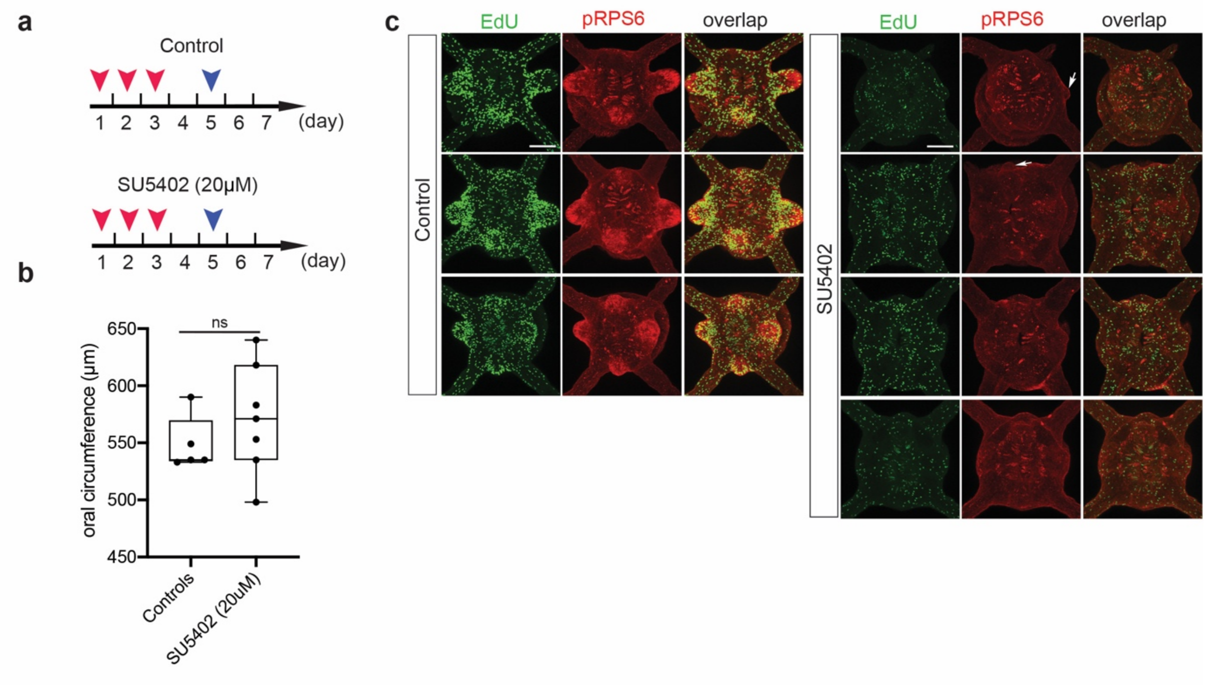

Supplementary Figure 11: **SU5402 inhibits localized growth in tentacle primordia. a** Feeding assay for controls and SU5402-treated polyps. Red arrowheads indicate the feeding days. Blue arrowheads show the days where animals were fixed. **b** Quantification of oral circumferences in the indicated conditions in two independent experiments. Data are shown as individual data points for each polyp in a Box and Whiskers graph (bottom: 25%; top: 75%; line: median; whiskers: min to max, unpaired Student's two-tailed t test *p*=0.36, ns: non-significant). **c** Confocal z-projections of oral poles of indicated polyps stained for EdU incorporation (green), an antibody against pRPS6 (two independent experiments). Arrows indicate reduced outgrowth forming in few SU5402-treated polyps. Scale bars are 50μm. Source data are provided as a Source Data file.

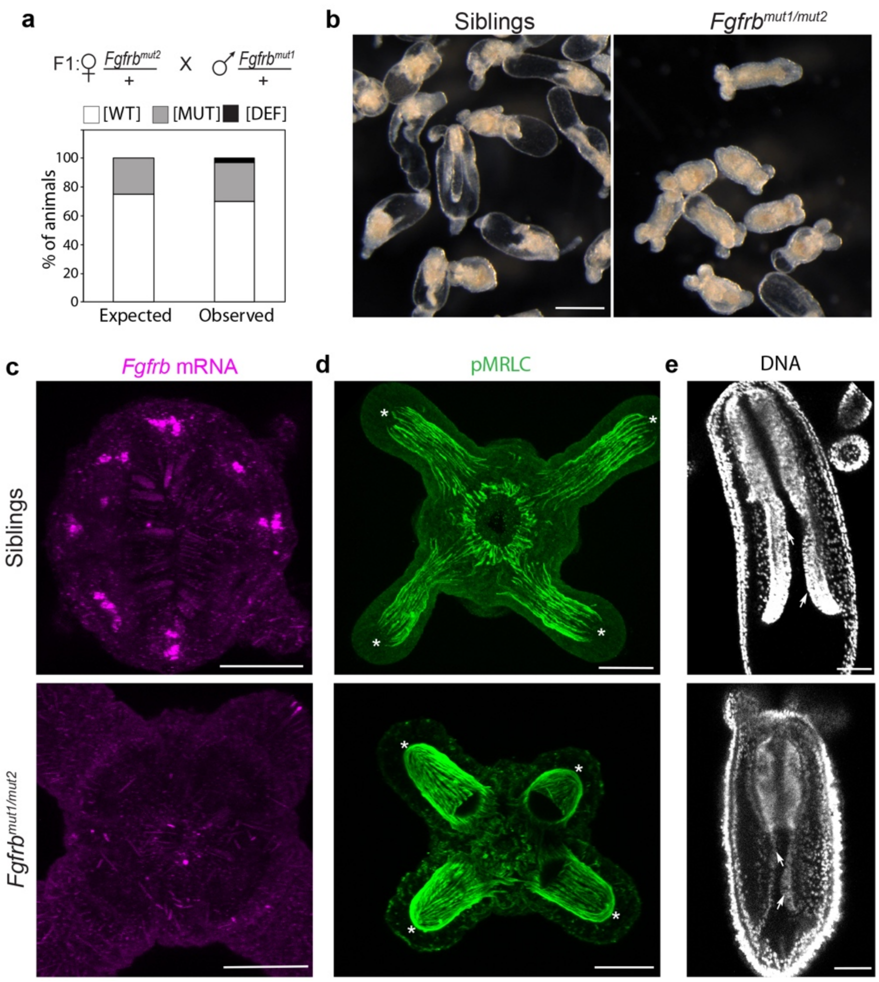

Supplementary Figure 12: **Phenotypic characterization of the *Fgfrb* mutants. a** F1 heterozygotes cross and quantification of F2 polyp phenotypes (n=156 polyps, three independent egg masses). These include wild type (WT), mutant (MUT) and deformity (DEF) phenotypes. **b** Images of *Fgfrb*$^{mut1/mut2}$ polyps and their siblings (three independent experiments). Scale bar is 250μm. **c** Fluorescent *in situ* hybridization of *Fgfrb* (purple) in wild type animals (n=6 polyps) and the *Fgfrb* mutants (n=5 polyps). **d** Immunostatining of phospho-Myosin Regulatory Chain (pMRC, green) labelling tentacular and oral muscles in a wild type and the *Fgfrb* mutant polyp (controls n=10 polyps, the *Fgfrb* mutant n=6 polyps, two independent experiments). Asterisks (*) indicate longitudinal muscle organization at the tentacle tips. Note that longitudinal muscles project through the tip region in the *Fgfrb* mutant while they do not cross in the wild type. **e**, Confocal z-projections of the body column of indicated polyps stained with Hoechst (white) (controls n=6 polyps, the *Fgfrb* mutant n=4 polyps, two independent experiments). White arrows show septal filaments. The *Fgfrb* mutant polyp has reduced septal filaments compared to the control. Scale bars are 50μm. Source data are provided as a Source Data file.
