# Supplementary material for: Feeding-dependent tentacle development in the sea anemone Nematostella vectensis
Source: Nat Commun. 2020 Sep 2;11:4399. doi: 10.1038/s41467-020-18133-0 (PMC7467937; doi:10.1038/s41467-020-18133-0)
Supplement: Supplementary file 6 — Reporting Summary [file 41467_2020_18133_MOESM6_ESM.pdf]

nature research

Corresponding author(s): Aissam Ikmi

Last updated by author(s): Jul 27, 2020

Reporting Summary

Nature Research wishes to improve the reproducibility of the work that we publish. This form provides structure for consistency and transparency in reporting. For further information on Nature Research policies, seeAuthors & Referees and theEditorial Policy Checklist .

Statistics

For all statistical analyses, confirm that the following items are present in the figure legend, table legend, main text, or Methods section.

n/a	Confirmed	
☐	☒	The exact sample size (*n*) for each experimental group/condition, given as a discrete number and unit of measurement
☐	☒	A statement on whether measurements were taken from distinct samples or whether the same sample was measured repeatedly
☐	☒	The statistical test(s) used AND whether they are one- or two-sided<br>*Only common tests should be described solely by name; describe more complex techniques in the Methods section.*
☒	☐	A description of all covariates tested
☒	☐	A description of any assumptions or corrections, such as tests of normality and adjustment for multiple comparisons
☒	☐	A full description of the statistical parameters including central tendency (e.g. means) or other basic estimates (e.g. regression coefficient) AND variation (e.g. standard deviation) or associated estimates of uncertainty (e.g. confidence intervals)
☐	☒	For null hypothesis testing, the test statistic (e.g. *F*, *t*, *r*) with confidence intervals, effect sizes, degrees of freedom and *P* value noted<br>*Give P values as exact values whenever suitable.*
☒	☐	For Bayesian analysis, information on the choice of priors and Markov chain Monte Carlo settings
☒	☐	For hierarchical and complex designs, identification of the appropriate level for tests and full reporting of outcomes
☒	☐	Estimates of effect sizes (e.g. Cohen's *d*, Pearson's *r*), indicating how they were calculated

Our web collection on statistics for biologists contains articles on many of the points above.

Software and code

Policy information about availability of computer code

Data collection	na
Data analysis	ImageJ (version:2.0.0-rc-69/1.52p) was used for image processing; CHOPCHOP v2: https://chopchop.cbu.uib.no was used to design sgRNAs; Imaris x64 v 9.2.1 was used for 3D image reconstruction. Prism 8 was used for all statistical analysis.

For manuscripts utilizing custom algorithms or software that are central to the research but not yet described in published literature, software must be made available to editors/reviewers. We strongly encourage code deposition in a community repository (e.g. GitHub). See the Nature Research guidelines for submitting code & software for further information.

Data

Policy information about availability of data

All manuscripts must include a data availability statement. This statement should provide the following information, where applicable:

- Accession codes, unique identifiers, or web links for publicly available datasets
- A list of figures that have associated raw data
- A description of any restrictions on data availability

We declare that the main data supporting the findings of this study are available within the article and its Supplementary Information files. Source data are available in the Source Data file for Figures 2,3,4,5,7, and Supplementary Figures 3, 5, 11, 12. Extra data are available from the corresponding author upon request.

Field-specific reporting

Please select the one below that is the best fit for your research. If you are not sure, read the appropriate sections before making your selection.

Life sciences study design

All studies must disclose on these points even when the disclosure is negative.

Sample size	No Sample size calculation was performed. Progeny from nine independent spawning events were segregated into three groups, each containing approximately 1500 animals. These large groups were then further subdivided into more manageable populations of 250-350 animals.
Data exclusions	No data were exluded
Replication	All experiments were repeated and the data presented in this study is based on at least two to three independent experiments. The number of repeats are given in the figure legends
Randomization	Animals were randomly allocated into experimental groups
Blinding	No Blinding was conducted. Experiments were performed by at least 2 independent investigators.

Reporting for specific materials, systems and methods

We require information from authors about some types of materials, experimental systems and methods used in many studies. Here, indicate whether each material, system or method listed is relevant to your study. If you are not sure if a list item applies to your research, read the appropriate section before selecting a response.

Materials & experimental systems

n/a	Involved in the study
☐	☒ Antibodies
☒	☐ Eukaryotic cell lines
☒	☐ Palaeontology
☐	☒ Animals and other organisms
☒	☐ Human research participants
☒	☐ Clinical data

Methods

n/a	Involved in the study
☒	☐ ChIP-seq
☒	☐ Flow cytometry
☒	☐ MRI-based neuroimaging

Antibodies

Antibodies used	Anti-pRPS6 Ser235/236 antibody (rabbit, dilution 1:50, Cell Signaling, clone D57.2.2E, #4858); rabbit anti-Phospho-Myosin Light Chain 2 (Ser19) Antibody (rabbit, dilution 1:50, Cell Signaling, # 3671); anti-eGFP (mouse, dilution 1:500, thermo fisher, clone 3E6, A-11120)
Validation	Anti-pRPS6 Ser235/236 antibody was validated by the vendor using Western blot analysis of extracts from PC12 and NIH/3T3 cells, treated with λ phosphatase, 20% FBS (20 min) or 100 ng/ml PDGF (20 min). We also validate this antibody in vivo by immunostaining experiments and Rapamycin treatment.<br>Anti-Phospho-Myosin Light Chain 2 (Ser19) was validated by the vendor using Western blot analysis of extracts from HeLa cells, vehicle-treated (-) or treated with the myosin light chain kinase inhibitor ML-7 (50µM, 15 min; +).<br>Anti-eGFP was validated by the vendor using immunostaining in HeLa cell transfected with pShooter pCMV/myc/mito/GFP, then fixed and permeabilized.

Animals and other organisms

Policy information about studies involving animals; ARRIVE guidelines recommended for reporting animal research

Laboratory animals	Males and females Laboratory strain of Nematostella vectensis were used in this study. The animals used in the experiments were between 2 to 3 weeks old.
Wild animals	No wild animals were used in the study.
Field-collected samples	no field-collected samples were used in the study.
Ethics oversight	Experiment involving cnidarians do not require ethics oversight.

Note that full information on the approval of the study protocol must also be provided in the manuscript.
