# Supplementary material for: Feeding-dependent tentacle development in the sea anemone Nematostella vectensis
Source: Nat Commun. 2020 Sep 2;11:4399. doi: 10.1038/s41467-020-18133-0 (PMC7467937; doi:10.1038/s41467-020-18133-0)
Supplement: Supplementary file 8 — Description of Additional Supplementary Files [file 41467_2020_18133_MOESM8_ESM.pdf]

Title: Supplementary Movie 1:

Description: 3D view of an oral pole showing *Fgfrb* expression (green) visualized by mRNA *in situ* hybridization and trained nuclei (blue). The 3D view was reconstructed using Imaris x64 9.2.1 (full ref: Imaris v 9.2.1, ImarisXT, Bitplane AG, software available at http://bitplane.com)

Title: Supplementary Movie 2:

Description: 3D view of the oral pole for the *Fgfrb-eGFP* transgenic line stained with α-eGFP (green) and Hoechst (blue).

Title: Supplementary Movie 3:

Description: ~Confocal z-stacks of the oral pole for a sibling polyp carrying the *Fgfrb-eGFP* transgene and stained with α-eGFP (green), phalloidin (red) and Hoechst (blue). Scale bar is 50μm.

Title: Supplementary Movie 4:

Description: Confocal z-stacks of the oral pole for an *Fgfrb* mutant polyp carrying the *Fgfrb-eGFP* transgene and stained with α-eGFP (green), phalloidin (red) and Hoechst (blue). Scale bar is 50μm.
